# A genome-wide association study identified a novel genetic loci *STON1-GTF2A1L/LHCGR/FSHR* for bilaterality of neovascular age-related macular degeneration

**DOI:** 10.1038/s41598-017-07526-9

**Published:** 2017-08-03

**Authors:** Kyoko Kawashima-Kumagai, Kenji Yamashiro, Munemitsu Yoshikawa, Masahiro Miyake, Gemmy Cheung Chui Ming, Qiao Fan, Jia Yu Koh, Masaaki Saito, Masako Sugahara-Kuroda, Maho Oishi, Yumiko Akagi-Kurashige, Isao Nakata, Hideo Nakanishi, Norimoto Gotoh, Akio Oishi, Hiroshi Tamura, Sotaro Ooto, Akitaka Tsujikawa, Yasuo Kurimoto, Tetsuju Sekiryu, Fumihiko Matsuda, Chiea-Chuen Khor, Ching-Yu Cheng, Tien Yin Wong, Nagahisa Yoshimura

**Affiliations:** 1 Department of Ophthalmology and Visual Sciences, Kyoto University Graduate School of Medicine, Kyoto, Japan; 2 Center for Genomic Medicine, Kyoto University Graduate School of Medicine, Kyoto, Japan; 3 Singapore Eye Research Institute, Singapore National Eye Centre, Singapore, Singapore; 4 Department of Ophthalmology, Fukushima Medical University, Fukushima, Japan; 5 Department of Ophthalmology, Kagawa University, Kagawa, Japan; 6 Department of Ophthalmology, Kobe City General Hospital, Kobe, Japan; 7 Saw Swee Hock School of Public Health, National University of Singapore and National University Health System, Singapore, Singapore; 8 Division of Human Genetics, Genome Institute of Singapore, Singapore, Singapore; 9 Ophthalmology and Visual Sciences Academic Clinical Program, Duke-NUS Graduate Medical School, National University of Singapore, Singapore, Singapore; 10 Department of Ophthalmology, Yong Loo Lin School of Medicine, National University of Singapore, Singapore, Singapore

## Abstract

Bilateral neovascular age-related macular degeneration (AMD) causes much more handicaps for patients than unilateral neovascular AMD. Although several AMD-susceptibility genes have been evaluated for their associations to bilaterality, genome-wide association study (GWAS) on bilaterality has been rarely reported. In the present study, we performed GWAS using neovascular AMD cases in East Asian. The discovery stage compared 581,252 single nucleotide polymorphisms (SNPs) between 803 unilateral and 321 bilateral Japanese cases but no SNP showed genome-wide significance, while SNPs at six regions showed *P*-value < 1.0 × 10^−5^, *STON1-GTF2A1L/LHCGR/FSHR, PLXNA1, CTNNA3*, *ARMS2/HTRA1*, *LHFP*, and *FLJ38725*. The first replication study for these six regions comparing 36 bilateral and 132 unilateral Japanese cases confirmed significant associations of rs4482537 (*STON1-GTF2A1L/LHCGR/FSHR*), rs2284665 (*ARMS2/HTRA1*), and rs8002574 (*LHFP*) to bilaterality. In the second replication study comparing 24 bilateral and 78 unilateral cases from Singapore, rs4482537 (*STON1-GTF2A1L/LHCGR/FSHR*) only showed significant association. Meta-analysis of discovery and replication studies confirmed genome-wide level significant association (*P* = 2.61 × 10^−9^) of rs4482537 (*STON1-GTF2A1L/LHCGR/FSHR*) and strong associations (*P* = 5.76 × 10^−7^ and 9.73 × 10^−7^, respectively) of rs2284665 (*ARMS2/HTRA1*) and rs8002574 (*LHFP*). Our GWAS for neovascular AMD bilaterality found new genetic loci *STON1-GTF2A1L/LHCGR/FSHR* and confirmed the previously reported association of *ARMS2/HTRA1*.

## Introduction

Age-related macular degeneration (AMD) is one of the major causes of visual impairment in developed countries. Although early stage AMD does not affect visual function, late stage AMD induces severe visual loss. AMD is a complex disease caused by multiple environmental and genetic risk factors. Previous genome-wide association studies (GWASs) identified two major susceptibility loci for AMD; complement factor H (*CFH*) and age-related maculopathy susceptibility 2/high temperature requirement A1 (*ARMS2/HTRA1)*. Recently, AMD Gene Consortium performed meta-analysis of GWASs and found 34 loci were associated with AMD development^1^. Furthermore, GWASs in East Asian populations revealed new loci for AMD and suggested ethnic differences in susceptibility to AMD^2, 3^.

Compared with patients with unilateral late AMD, patients with bilateral late AMD are more prone to visual handicaps. The prevalence of bilateral AMD was reported to be 40–50% in Caucasian and 10–20% in Asian^4–10^. Previous studies investigated the association between bilaterality of AMD and the known AMD susceptibility loci. Several studies reported that *ARMS2/HTRA1* contributes to the bilaterality of late AMD^10–16^. In contrast, it is still controversial whether *CFH* increases the risk of AMD bilaterality^10, 12–14, 16–19^. To identify the genetic determinants associated with bilaterality of late AMD in East Asian, we conducted a GWAS comparing bilateral late AMD patients with unilateral late AMD patients. Since most late AMD is neovascular AMD (wet type) in East Asian and geographic atrophy (dry type) is rare, we focused on only neovascular AMD. After finding genes associated with the bilaterality of neovascular AMD, we confirmed their susceptibility to AMD occurrence by comparing all neovascular AMD cases including both bilateral cases and unilateral cases with controls of Japanese general populations.

## Results

Japanese patients with neovascular AMD were recruited at the Center for Macular Diseases of Kyoto University Hospital (n = 821) and Fukushima Medical University (n = 333) for the discovery stage. From these 1154 cases, 10 cases were excluded due to lack of detailed fundus examination of the fellow eye and 20 cases were excluded because of the quality control for their genotype count analysis. Of the 1124 cases analyzed in the discovery stage, 803 had unilateral neovascular AMD and 321 had bilateral neovascular AMD. Subtypes of AMD were typical AMD in 507 patients and PCV in 617 patients. Clinical features of subjects are summarized in Table 1. Patients with bilateral neovascular AMD were older than patients with unilateral neovascular AMD (*P < *0.001). While 70–80% of patients were male, the percentages were similar between bilateral and unilateral groups. The number of SNPs evaluated after quality control was 581,252. We plotted our genome-wide association findings on quantile-quantile (QQ) plots, and genomic control method revealed only a slight inflation of the test statistics (Inflation factor λ = 1.01).

**Table 1:** Neovascular age-related macular degeneration samples used in the study

	Bilateral neovascular AMD			Unilateral neovascular AMD			P-value	
	n	Age	Sex (%, male)	n	Age	Sex (%, male)	Age	Sex
**Discovery stage**								
Kyoto	247	80.9 ± 7.8	70.5	546	76.4 ± 8.4	69.6	<0.001	0.81
Fukushima	74	81.6 ± 6.3	83.8	257	77.6 ± 7.9	75.1	<0.001	0.12
Total	321	81.1 ± 7.4	73.5	803	76.8 ± 8.3	71.4	<0.001	0.47
**Replication stage**								
Kobe	36	83.4 ± 7.0	77.8	132	77.4 ± 8.0	71.2	<0.001	0.43
Singapore	24	71.6 ± 5.9	70.8	78	66.0 ± 10.2	61.5	0.0013	0.56

**Figure 1:**
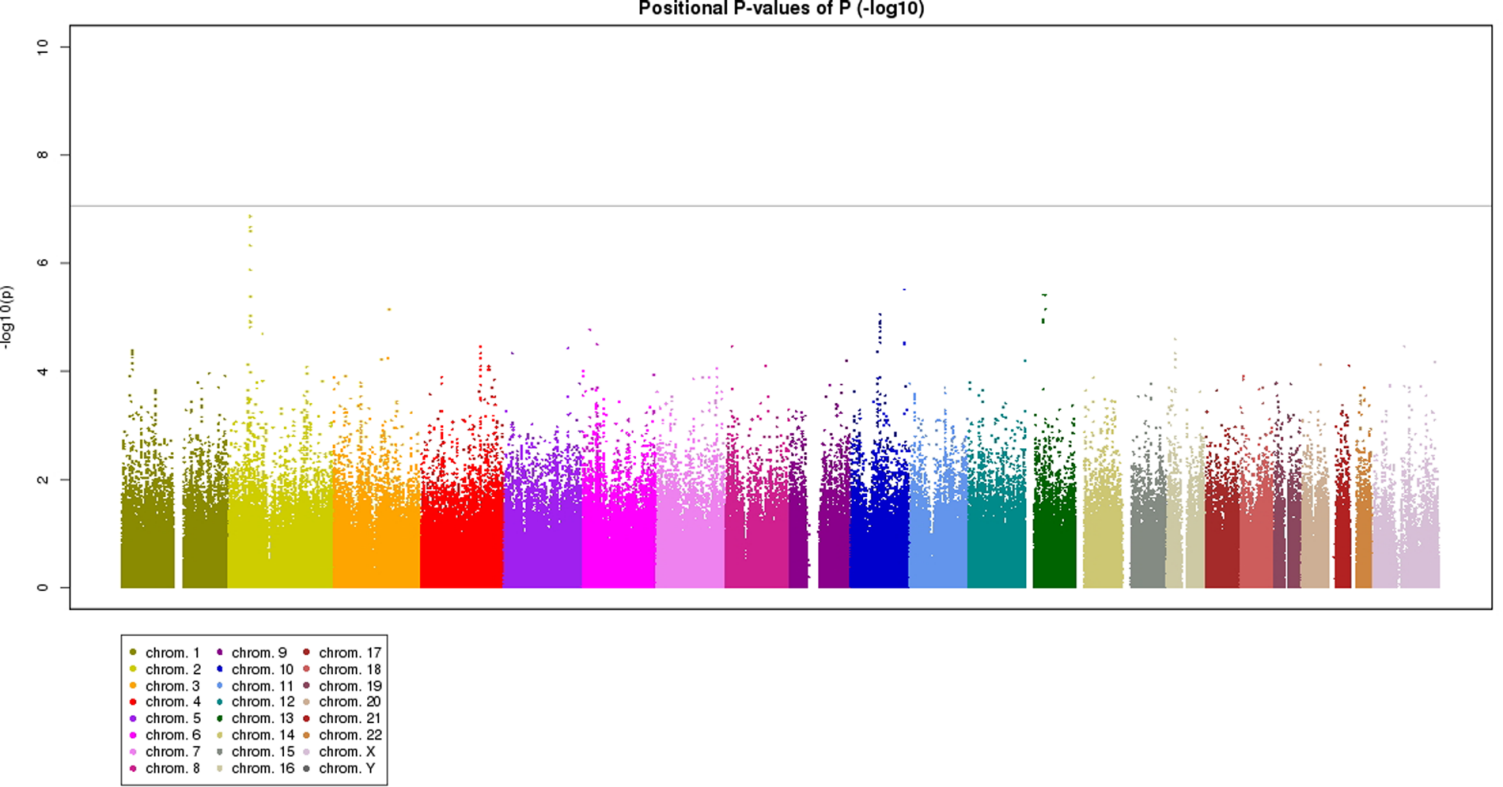
Minus log-transformed P-values are shown in a signal intensity (Manhattan) plot relative to their genomic position for bilaterality of age-related macular degeneration. P-values are adjusted for age and sex.

Results of the genome-wide association analysis are shown in Fig. 1. Although no chromosomal loci showed genome-wide significance, fourteen SNPs showed *P*-value < 1.0 × 10^−5^; 7 SNPs in *STON1-GTF2A1L/LHCGR/FSHR* region, 1 SNP in *PLXNA1*, 2 SNPs in *CTNNA3*, 1 SNP in *ARMS2/HTRA1*, 1 SNP in *LHFP*, and 2 SNPs near *FLJ38725* (Table 2). No SNPs in *CFH* showed significant association with bilaterality of neovascular AMD (*P* > 0.05). Supplementary Table 1 shows the association between the AMD bilaterality and the analyzed 440 SNPs within 10 genes for which associations with AMD were verified in Asian individuals. Although rs11963725 of *C2/CFB* (*P* = 0.0124), rs1054060 of C3 (*P* = 0.0454), rs17310296 of *CETP* (*P* = 0.00250), rs4714699 of *VEGFA* (*P* = 0.0368), rs6822976 of *CFI* (*P* = 0.00256), and rs12638651 of *ADAMTS9* (*P* = 0.00742) showed nominally significant associations, these associations should be interpreted as negative results after permutation tests.

**Table 2:** Results of discovery study for the 14 SNPs that showed *P*-value < 10^−5^, comparing bilateral neovascular age-related macular degeneration cases with unilateral those.

SNP	Chr	Nearest Gene	Allele		Bilateral neovascular AMD				Unilateral neovascular AMD				*P*-value	OR (95% CI)	*P*-value*	OR (95% CI)*
			1	2	11	12	22	MAF	11	12	22	MAF				
rs7589251	2	*STON1- GTF2A1L/LHCGR/FSHR*	G	T	52	133	136	0.37	58	321	423	0.27	6.22 × 10^−6^	1.56 (1.28–1.91)	4.16 × 10^−6^	1.60 (1.31–1.96)
rs10208693	2		A	G	51	138	132	0.37	56	314	433	0.27	3.53 × 10^−7^	1.65 (1.35–2.01)	4.74 × 10^−7^	1.68 (1.37–2.05)
rs4538253	2		G	A	58	139	124	0.40	69	344	390	0.30	9.49 × 10^−6^	1.54 (1.26–1.88)	9.55 × 10^−6^	1.56 (1.28–1.90)
rs7603311	2		T	C	48	137	134	0.37	54	311	438	0.26	9.14 × 10^−7^	1.63 (1.33–2.00)	1.35 × 10^−6^	1.65 (1.35–2.02)
rs4482537	2		C	T	52	142	121	0.39	60	326	411	0.28	3.78 × 10^−7^	1.65 (1.34–2.02)	1.36 × 10^−7^	1.73 (1.41–2.12)
rs6545074	2		G	T	50	142	129	0.38	54	326	420	0.27	8.23 × 10^−7^	1.63 (1.32–2.00)	2.54 × 10^−7^	1.71 (1.39–2.10)
rs7037739	2		G	A	48	141	132	0.37	48	325	430	0.26	4.73 × 10^−7^	1.65 (1.34–2.02)	2.14 × 10^−7^	1.73 (1.40–2.12)
rs900429	3	*PLXNA1*	C	T	8	98	214	0.18	45	329	428	0.26	2.96 × 10^−5^	0.61 (0.48–0.78)	7.18 × 10^−6^	0.57 (0.45–0.73)
rs1925616	10	*CTNNA3*	G	A	24	129	168	0.28	25	263	515	0.19	2.77 × 10^−5^	1.57 (1.25–1.97)	8.79 × 10^−6^	1.67 (1.33–2.10)
rs10997482	10		A	G	24	129	168	0.28	25	263	515	0.19	2.77 × 10^−5^	1.57 (1.25–1.97)	8.79 × 10^−6^	1.67 (1.33–2.10)
rs2284665	10	*ARMS2/HTRA1*	G	T	43	99	179	0.29	147	359	296	0.41	1.39 × 10^−7^	0.59 (0.48–0.71)	3.08 × 10^−6^	0.63 (0.52–0.76)
rs8002574	13	*LHFP*	T	C	2	33	285	0.06	12	177	614	0.13	2.86 × 10^−6^	0.43 (0.29–0.63)	3.84 × 10^−6^	0.41 (0.28–0.60)
rs9525873	13	*FLJ38725*	T	C	49	172	100	0.42	97	327	378	0.32	1.77 × 10^−5^	1.51 (1.25–1.84)	7.13 × 10^−6^	1.56 (1.29–1.90)
rs895266	13		T	C	74	168	79	0.49	135	362	305	0.39	2.07 × 10^−5^	1.49 (1.23–1.80)	3.86 × 10^−6^	1.57 (1.29–1.90)

SNP, single nucleotide polymorphism; Chr, chromosome; MAF, minor allele frequency; OR, odds ratio; CI, confidence interval.

*Adjusted for age and sex.

One representative SNP from each of these six loci was examined in the replication studies (Table 3). For the first replication stage, Japanese patients with neovascular AMD were recruited at Kobe City Medical Center General Hospital (n = 170). Two cases were excluded after quality control for the genotype count analysis, and the genotypes of 36 bilateral AMD and 132 unilateral AMD were compared. The associations with neovascular AMD bilaterality were confirmed in rs4482537 of *STON1-GTF2A1L/LHCGR/FSHR* (*P* = 4.97 × 10^−2^), rs2284665 of *ARMS2/HTRA1* (*P* = 3.83 × 10^−2^), and rs8002574 of *LHFP* (*P* = 3.66 × 10^−2^). For the second replication stage, Singaporean patients with neovascular AMD (n = 102) were recruited at Singapore National Eye Center. In the second replication study with 24 bilateral and 78 unilateral cases, only rs4482537 of *STON1-GTF2A1L/LHCGR/FSHR* showed significant association (*P* = 3.34 × 10^−2^), while rs2284665 of *ARMS2/HTRA1* and rs8002574 of *LHFP* showed same association direction as in the discovery stage analysis and the first replication study with Japanese without statistically significant *P*-value. The meta-analysis of discovery and replication studies confirmed genome-wide significant association of *STON1-GTF2A1L/LHCGR/FSHR* (rs4482537; *P* = 2.61 × 10^−9^) with bilaterality. As for *ARMS2/HTRA1* and *LHFP*, the effect direction was same among three cohorts and meta-analysis further confirmed their strong associations with bilaterality (*P* = 5.76 × 10^−7^ for rs2284665 of *ARMS2/HTRA1* and *P* = 9.73 × 10^−7^ for rs8002574 of *LHFP)*.

**Table 3:** Results of two replication studies and meta-analysis for the six loci associated in discovery study, comparing bilateral neovascular age-related macular degeneration cases with unilateral those.

SNP	Nearest Gene	Replication study								Discovery and replication study			
		Kobe (n = 168)				Singapore (n = 102)				Combined meta-analysis			
		*P* value	OR (95% CI)	*P* value*	OR (95% CI)	*P* value	OR (95% CI)	*P* value*	OR (95% CI)*	*P* value	OR(95% CI)	*P* value*	OR (95% CI)*
rs4482537	*STON1- GTF2A1L/ LHCGR/ FSHR*	0.048	1.71 (1.01–2.90)	0.050	1.78 (1.00–3.16)	0.034	2.42 (1.07–5.49)	0.033	2.53 (1.08–5.93)	2.88 × 10^−8^	1.69 (1.40–2.03)	2.61 × 10^−9^	1.76 (1.46–2.13)
rs2284665	*ARMS2/HTRA1*	0.002	0.39 (0.21–0.72)	0.038	0.52 (0.28–0.97)	0.601	0.83 (0.42–1.66)	0.711	0.88 (0.43–1.77)	1.65 × 10^−9^	0.58 (0.49–0.69)	5.76 × 10^−7^	0.63 (0.53–0.76)
rs8002574	*LHFP*	0.124	0.47 (0.16–1.36)	0.037	0.32 (0.11–0.93)	0.801	0.89 (0.38–2.13)	0.584	0.78 (0.32–1.90)	1.60 × 10^−5^	0.48 (0.35–0.67)	9.73 × 10^−7^	0.44 (0.32–0.61)
rs895266	*FLJ38725*	0.726	0.91 (0.53–1.56)	0.552	0.85 (0.49–1.46)	0.353	0.72 (0.38–1.43)	0.364	0.72 (0.36–1.46)	8.86 × 10^−4^	1.35 (1.13–1.61)	1.40 × 10^−4^	1.40 (1.18–1.67)
rs900429	*PLXNA1*	0.423	1.28 (0.67–2.43)	0.367	1.34 (0.71–2.54)	0.133	1.71 (0.85–3.44)	0.235	1.57 (0.75–3.28)	4.84 × 10^−3^	0.73 (0.59–0.91)	8.46 × 10^−4^	0.69 (0.55–0.86)
rs1925616	*CTNNA3*	0.822	0.93 (0.46–1.90)	0.928	1.03 (0.51–2.10)	—	—	0.709	0.85 (0.37–1.96)	—	—	5.59 × 10^−5^	1.54 (1.25–1.90)

SNP, single nucleotide polymorphism; OR, odds ratio; CI, confidence interval.

*Adjusted for age and sex.

To evaluate the associations of these three SNPs with the occurrence of AMD, genotypes of all neovascular AMD cases including both bilateral cases and unilateral cases were compared with the Nagahama cohort as Japanese general control (Table 4). The analysis showed that only *ARMS2/HTRA1* was significantly associated with the occurrence of AMD (*P* = 1.78 × 10^−81^).

**Table 4:** Results of the association study for three loci about the occurrence of AMD, comparing all neovascular age-related macular degeneration cases with Japanese general population cohort.

SNP	Allele		Control (Nagahama cohort, n = 3265)					AMD cases (n = 1394)				*P* -value* (OR, 95%CI)
	1	2	11	12	22	AF1	11		12	22	MAF	
rs4482537 *(LHCGR/FSHR)*	C	T	280	1430	1555	0.30	134		582	666	0.31	0.79 (1.01, 0.92–1.12)
rs2284665 *(ARMS2/HTRA1)*	G	T	1165	1556	544	0.60	232		567	594	0.37	1.78 × 10^−81^ (0.40, 0.36–0.44)
rs8002574 *(LHFP)*	T	C	53	690	2522	0.12	21		265	1107	0.11	0.11 (0.89, 0.78–1.03)

SNP, single nucleotide polymorphism; AF, allele frequency; MAF, minor allele frequency; OR, odds ratio; CI, confidence interval, *Chi-trend.

## Discussion

In this current study, we performed GWAS on bilaterality of neovascular AMD for the first time and identified the association of new genetic loci *STON1-GTF2A1L/LHCGR/FSHR* with neovascular AMD bilaterality. ARMS2/*HTRA1* could be also associated with bilaterality of neovascular AMD and the association of *LHFP* should be further investigated.

Regional association plot (Supplementary Figure 1) for the studied SNPs within *STON1-GTF2A1L/LHCGR/FSHR* region did not elucidate which of these genes was correspond to the bilaterality of AMD. *STON1* encodes stonin 1. Although stonin 2 controls synaptic transmission, the function of stonin 1 has not been elucidated^20^. *GTF2A1L* is expressed mainly in testis to form a counterpart of general transcription factor IIA subunit. In drosophila, transcription factor IIA regulates development of photoreceptor cells^21^. Polymorphisms in *STON1-GTF2A1L* might promote bilateral development of AMD through their effects on photoreceptor cells synaptic transmission.

*LHCGR* encodes luteinizing hormone/choriogonadotropin receptor, a receptor for luteinizing hormone (LH) and human chorionic gonadotropin (hCG), and *FSHR* encodes follicle-stimulating hormone receptor, a receptor for follicle-stimulating hormone (FSH). Both LH and FSH are released from the pituitary gland and stimulate estrogen secretion. Estrogen is associated with inhibition of AMD development^22–24^ and a previous study reported that polymorphisms of estrogen receptor gene were associated with AMD^25^. Aging decreases estrogen production, leading to increased LH/FSH secretion in the elderly via feedback mechanism.

Age-related decrease in estrogen production and increase in LH/FSH secretion are associated, partly, with increased risk of atherosclerosis and heart diseases by affecting lipoprotein/cholesterol metabolism^26–28^. Considering that several genes in lipoprotein/cholesterol metabolism are associated with AMD development, *LHCGR/FSHR* would affect the bilaterality of neovascular AMD in part by altering lipoprotein/cholesterol metabolism. Another possibility is that *LHCGR/FSHR* polymorphism would have localized effect, thereby affecting neovascular AMD bilaterality. Müller cells and retinal pigmented epithelial cells produce hCG and cone photoreceptor cells express its receptor LHCGR^29^. Further study on the roles of LHCGR of photoreceptor cells and the roles of *LHCGR/FSHR*-induced lipoprotein/cholesterol metabolism alteration would lead to prevention of neovascular AMD development in the fellow eye.

*STON1-GTF2A1L/LHCGR/FSHR* region also includes long intergenic noncoding RNA (lincRNA, RP11-460M2.1). LincRNA can control gene expression and some lincRNAs might be able to control ocular neovascularization^30^. LincRNA RP11-460M2.1 might have some ability to control bilaterality of neovascular AMD.

Although *CFH* is a major susceptibility gene for AMD, SNPs in *CFH* did not have any association with the bilaterality of neovascular AMD in our study. Previous studies also showed that *CFH* was not associated with the bilaterality of neovascular AMD^4, 7, 10, 11^. In contrast, rs4482537 in *LHCGR/FSHR* locus had significant association with the bilaterality of neovascular AMD, but not with the occurrence of AMD. On the other hand, previous studies and current study support that *ARMS2/HTRA1* is associated with both occurrence and bilaterality of AMD. AMD-associated genes can be classified into three types, genes associated with both AMD occurrence and bilaterality, genes associated with only AMD occurrence, and genes associated with only AMD bilaterality. The second eye involvement in AMD might be regulated by a unique mechanism in addition to the factors associated with the first eye involvement.

*LHFP* is a HMGIC fusion partner gene in lipoma, one of the most common mesenchymal tumors^31^. *LHFP* is also associated with mesenchymal differentiation in gliosarcoma^32^. Recent studies suggest that epithelial–mesenchymal transition (EMT) has important roles in the development of AMD^33, 34^. *LHFP* might affect the bilaterality of neovascular AMD by affecting the EMT process. *LHFP* is also associated with Alzheimer’s disease^35^ that shares common clinical and pathological features with AMD; both Alzheimer’s disease and AMD are preceded by accumulation of amyloid beta^36^. *LHFP* might affect accumulation of amyloid beta and trigger the second eye involvement of AMD.

Although anti-VEGF treatment has improved treatment outcome of exudative AMD, the SEVEN-UP study reported that 51% of the patients in their study suffered from bilateral neovascular AMD during 7 years of follow-up^37^. The second eye involvement in patients with late AMD is a matter of concern because patients with bilateral late AMD are more prone to visual handicaps than patients with unilateral late AMD. The SEVEN-UP study also suggested that reduced frequency of treatments contributed to the decline of visual acuity. Increasing the frequency of injection might maintain visual acuity for an extended period. Therefore intensive treatment should be initiated in patients with unilateral neovascular AMD who are most likely to develop bilateral neovascular AMD in the future, to maintain good visual acuity. Prediction of the second eye involvement in neovascular AMD would be beneficial for patients with unilateral neovascular AMD. Elucidation of the mechanism to control the second eye involvement might lead to prevention of the second eye involvement.

The limitations of this study are its retrospective nature and relatively small sample size. Studies involving large size might successfully replicate the association of *LHFP*, and prospective study would further confirm the association of *STON1-GTF2A1L/LHCGR/FSHR* and *ARMS2/HTRA1* to the bilaterality of AMD. Considering that CATT study could not detect any genetic associations between polymorphisms and the second eye involvement within 2 years of follow-up^17^, studies with longer follow-up period should be performed. The unilateral patients included in the current study were significantly younger than the bilateral patients. The unilateral patients may go on to develop bilateral disease with longer follow up, which would reduce the power of detecting genetic associations. Statistical adjustment for the time of diagnosis with unilateral neovascular AMD would also be helpful. Current study was performed only in Asians including both typical AMD and PCV. The prevalence of PCV is higher in Asian than Caucasian^4–10, 38^. Although the reported prevalence of bilateral involvement is similar between typical AMD and PCV, the ethnic difference cannot be ignored; 40–50% in Caucasian^4, 5^ and 10–20% in Asian^6–10^. Recent studies also suggested the role of ethnic differences in AMD genetic susceptibility^3^. Further study is needed to evaluate whether *STON1-GTF2A1L/LHCGR/FSHR* and *LHFP* are associated with neovascular AMD bilaterality in other races.

In conclusion, our GWAS for neovascular AMD bilaterality identified novel genetic loci *STON1-GTF2A1L/LHCGR/FSHR* and confirmed the association of *ARMS2/HTRA1* with the bilaterality. *LHFP* might also be associated with AMD bilaterality. Prediction of the second eye involvement would be beneficial in determining the long-term management strategy for the first eye of AMD, and elucidation of the mechanisms for the second eye involvement would lead to prevention of the second eye involvement in AMD.

## Methods

All procedures used in this study confirmed to the tenets of the Declaration of Helsinki. The Institutional Review Board and the Ethics Committee of each institution approved the experimental protocols; The Kyoto University Graduate School and Faculty of Medicine Ethics Committee, Fukushima Medical University Ethics Committee, Kobe City Medical Center General Hospital Ethics Committee, Singapore National Eye Center Ethics Committee, the Ad hoc Review Board of the Nagahama Cohort Project, and the Nagahama Municipal Review Board of Personal Information Protection. All the participants were fully informed of the purpose and procedures and a written consent was obtained from each.

### Study subjects in GWAS for bilaterality

Japanese patients with neovascular AMD were recruited at the Center for Macular Diseases of Kyoto University Hospital (n = 821) and Fukushima Medical University (n = 333) for the discovery stage, and at Kobe City Medical Center General Hospital (n = 170) for the replication stage. Further, patients with neovascular AMD (n = 112) were recruited at Singapore National Eye Center for the second replication stage. All subjects underwent comprehensive ophthalmologic examinations, including dilated contact lens slit-lamp biomicroscopy, fundus photography, fluorescein and indocyanine green angiography (HRA2, Heidelberg Engineering, Heidelberg, Germany), and optical coherence tomography (Spectralis HRA + OCT, Heidelberg Engineering, Heidelberg, Germany).

Neovascular AMD was defined as the presence of exudative AMD as described in the international classification system for age-related maculopathy. Typical AMD involved classic choroidal neovascularization (CNV), occult CNV, or a combination of both. The diagnosis of polypoidal choroidal vasculopathy (PCV) was based on indocyanine green angiography, which showed a branching vascular network terminated in polypoidal lesions. Diagnosis and grading of AMD were performed in a masked manner by two ophthalmologists independently. In cases of disagreement, the third retinal specialist made the final decision. Bilaterality of neovascular AMD and subject age at final visit was used for analysis.

### Genotyping

Genomic DNAs were extracted from peripheral blood leukocytes using QuickGene-610L DNA extraction kit (FUJIFILM Co., Tokyo, Japan). Genotyping was performed using Illumina BeadChip, both OmniExpress and HumanExome, HumanOmni2.5–8, or OmniExpress. The distortion of Hardy-Weinberg equilibrium (HWE) was not considered in this study because all samples comprised AMD cases. Stringent quality control, including minor allele frequency (MAF) ≥ 1% and genotype call rate ≥ 95% (per SNP and per individual), was performed using PLINK ver1.07 (http://pngu.mgh.harvard.edu/~purcell/plink/).

### Statistical analysis

Association between genotypic distribution of each SNP and the bilaterality of neovascular AMD was examined using logistic regression analysis by adjusting for age and sex using Software R (R Foundation for Statistical Computing, Vienna, Austria). Inflation of the test statistics was assessed using the genomic-control method. SNPs with *P*-value < 1.0 × 10^−5^ were selected as candidates of replication stages. Among the candidate SNPs, one representative SNP was selected from each locus (r^2^ > 0.8 in the discovery stage samples) and these SNPs were tested for association in replication stages. The genotypic counts of the first and second stages were also evaluated in meta-analysis.

Furthermore, association between genotypic distribution of each candidate SNP of neovascular AMD bilaterality and the occurrence of neovascular AMD was examined by chi-square test for trend. All neovascular AMD cases were compared with Japanese general cohort, the Nagahama Study^39^, as control.

### Control cohort for the AMD susceptibility test

A fixed dataset of 3,265 unrelated healthy Japanese subjects from the Nagahama prospective genome cohort for the Comprehensive Human Bioscience (The Nagahama Study) was used as a control group in the AMD susceptibility test. In detail, a total of 3,712 individuals from the Nagahama study were genotyped using HumanHap610K Quad Arrays, HumanOmni2.5 M Arrays, and/or HumanExome Arrays (Illumina Inc., CA, USA). To ensure high-quality genotype data, a series of quality control filters were applied to the data from each platform before imputation, including MAF cut-offs (>0.01), HWE (*P* > 1.0 × 10^−6^), genotypic success rate (>95%), individual call rate (>99%), and estimated relatedness (PI-HAT < 0.35). Quality controls were performed using PLINK (ver.1.07; http://pngu.mgh.harvard.edu/,purcell/plink/). For consistent genotyping data across each platform, we performed genomic imputation on available 1000 Genome Project data from East Asian subjects using MACH software (http://www.sph.umich.edu/csg/abecasis/MACH/tour/imputation.html). After imputation, we again performed quality control including MAF cut-offs (>0.01), HWE (*P* > 1.0 × 10^−7^), genotypic success rate (>90%), individual call rate (>90%), and imputation quality (R2 > 0.3).

## Electronic supplementary material

Dataset 1

Dataset 2
