# Supplementary material for: A genome-wide association study identified a novel genetic loci STON1-GTF2A1L/LHCGR/FSHR for bilaterality of neovascular age-related macular degeneration
Source: Sci Rep. 2017 Aug 3;7:7173. doi: 10.1038/s41598-017-07526-9 (PMC5543064; doi:10.1038/s41598-017-07526-9)

**A genome-wide association study identified a novel genetic loci *STON1-GTF2A1L/LHCGR/FSHR* for bilaterality of neovascular age-related macular degeneration**

Kyoko Kawashima-Kumagai, Kenji Yamashiro, Munemitsu Yoshikawa, Masahiro Miyake, Gemmy Cheung Chui Ming, Qiao Fan, Jia Yu Koh, Masaaki Saito, Masako Sugahara-Kuroda, Maho Oishi,Yumiko Akagi-Kurashige, Isao Nakata, Hideo Nakanishi, Norimoto Gotoh, Akio Oishi, Hiroshi Tamura, Sotaro Ooto, Akitaka Tsujikawa, Yasuo Kurimoto, Tetsuju Sekiryu, Fumihiko Matsuda, Chiea-Chuen Khor, Ching-Yu Cheng, Tien Yin Wong, Nagahisa Yoshimura

**Supplementary Figure 1.** Regional association plot for *STON1-GTF2A1L/LHCGR/FSHR* region


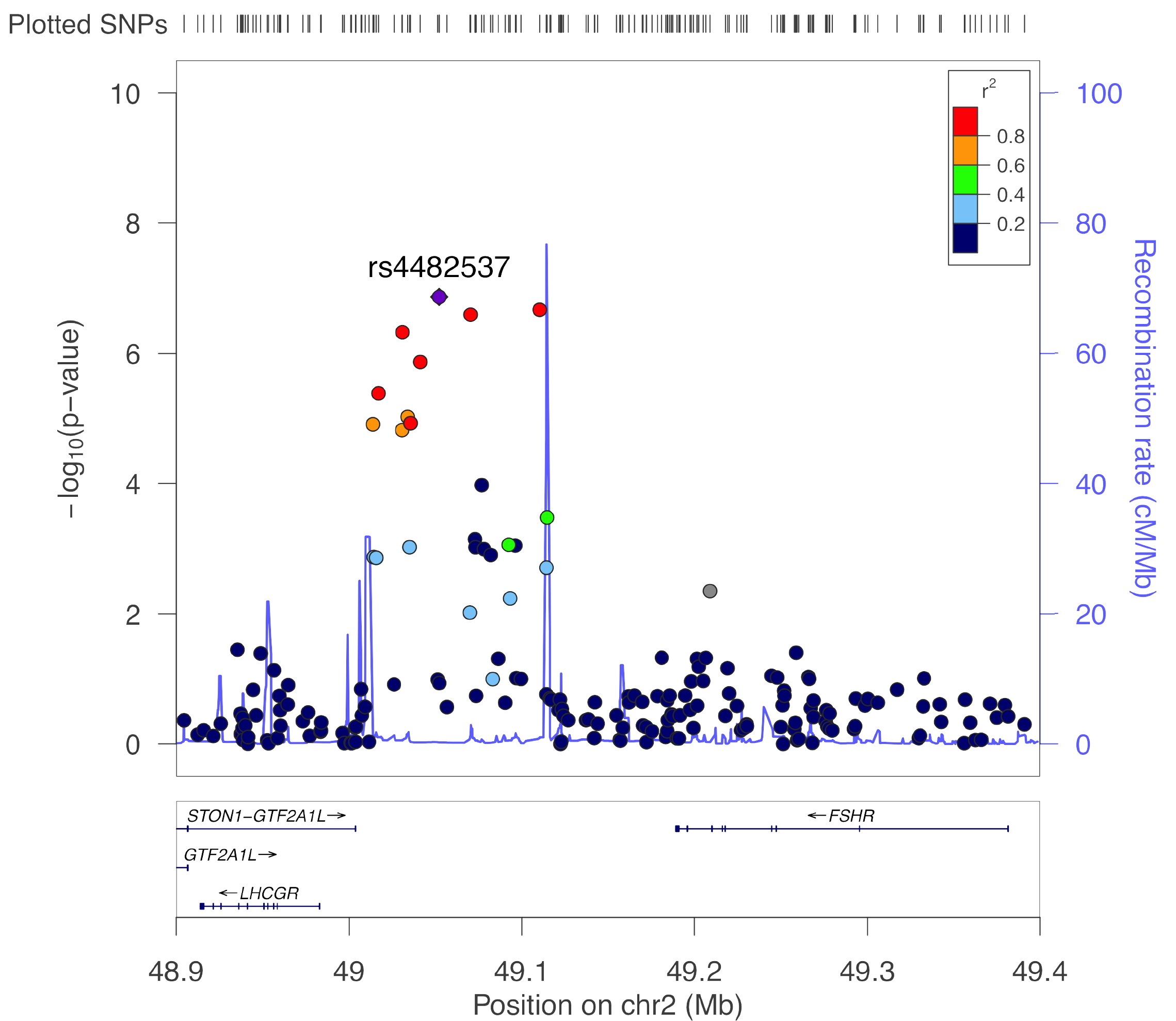

Supplement: Supplementary file 2 — Dataset 2 [file 41598_2017_7526_MOESM2_ESM.doc]
